# Supplementary material for: TERT Promoter Mutations and the 8th Edition TNM Classification in Predicting the Survival of Thyroid Cancer Patients
Source: Cancers (Basel). 2021 Feb 5;13(4):648. doi: 10.3390/cancers13040648 (PMC7915040; doi:10.3390/cancers13040648)
Supplement: Supplementary file 1 [file cancers-13-00648-s001.pdf]

Supplementary Materials: TERT Promoter Mutations and the Eighth Edition TNM Classification in Predicting the Survival of Thyroid Cancer Patients

Jun Park, Sungjoo Lee, Kyunga Kim, Hyunju Park, Chang-Seok Ki, Young Lyun Oh, Jung Hee Shin, Jee Soo Kim, Sun Wook Kim, Jae Hoon Chung and Tae Hyuk Kim

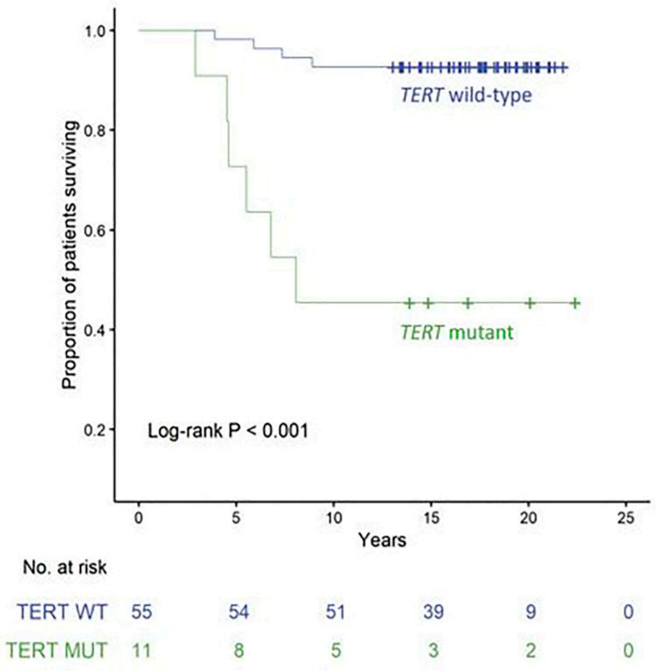

Figure S1. Kaplan–Meier curves of thyroid cancer-specific survival according to TERT promoter mutational status in patients with follicular thyroid cancer.

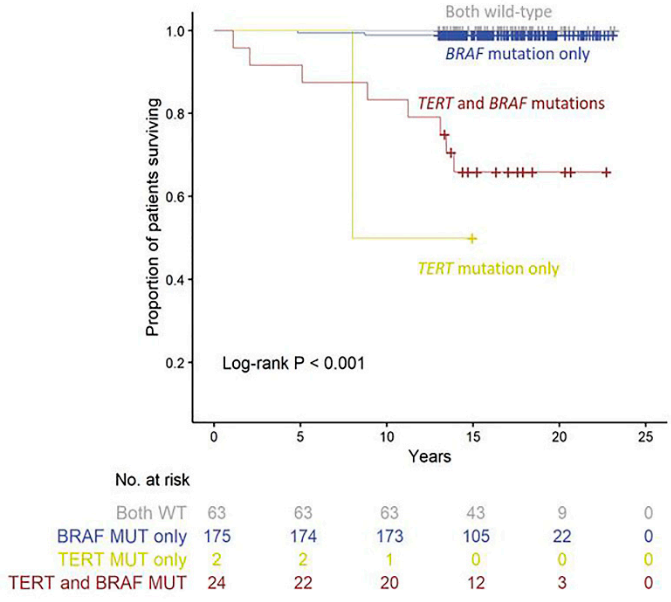

Figure S2. Kaplan–Meier curves of thyroid cancer-specific survival according to a combination of TERT promoter mutation and BRAF mutation statuses in patients with papillary thyroid cancer. Patients with BRAF mutations only had a 10-year survival rate of 98.9%, whereas patients with both TERT and BRAF mutations had a 10-year survival rate of 83.3%.

Table S1. Subgroup analysis of stage II patients according to downstaging by TNM-8.

N, (%)	Group 1[a] (*n* = 10)	Group 2[b] (*n* = 38)	*p*-value
MUT TERT	1 (10)	16 (42.1)	0.074
Cancer-specific death	1 (10)	10 (26.3)	0.416
Cancer-specific death with MUT TERT	0	8 (80.0)	0.273

[a] Group 1: TNM-8 Stage II patients who were also Stage II in TNM-7; [b] Group 2: TNM-8 Stage II Patients who were downstaged by TNM-8; Abbreviations: TNM-8, the American Joint Committee on Cancer Thyroid Cancer Staging System 8th edition; MUT, mutant; TERT, telomerase reverse transcriptase.

Table S2. Subgroup analysis of downstaged stage II patients according to the cause of downstaging in TNM-8.

N, (%)	Group 2A[a] (*n* = 6)	Group 2B[b] (*n* = 32)	*p*-value
MUT TERT	3 (50)	13 (40.6)	0.682
Cancer-specific death	4 (66.7)	6 (18.8)	0.031
Cancer-specific death with MUT TERT	3 (75)	5 (83.3)	1.000

[a] Group 2A: TNM-8 Stage II Patients who were downstaged by TNM-8 according to age; [b] Group 2B: TNM-8 Stage II Patients who were downstaged by TNM-8 according to tumor classification; Abbreviations: TNM-8, the American Joint Committee on Cancer Thyroid Cancer Staging System 8th edition; MUT, mutant; TERT, telomerase reverse transcriptase.

Table S3. Associations between *TERT* mutation status or clinicopathological variables (including total RAI dose) and thyroid cancer-specific survival in patients with DTC.

Variables	N[a]	10-year survival rate (%)	Univariate Cox models		Multivariate Cox model 1[b] (extended model)		Multivariate Cox model 2[c] (restricted model)	
			HR (95% CI)	*p*-value	HR (95% CI)	*p*-value	HR (95% CI)	*p*-value
Sex	-	-	-	-	-	-	-	-
Female	329	95.1	1.00 (reference)	0.167	1.00 (reference)	0.071	1.00 (reference)	0.105
Male	64	92.2	1.83 (0.78–4.34)	-	2.38 (0.93–6.08)	-	2.19 (0.85–5.64)	-
Age, years[d]	-	-	-	-	-	-	-	-
Per 5 years	393		1.75 (1.50–2.06)	< 0.001	1.48 (1.20–1.83)	< 0.001	-	-
< 55	319	97.5	1.00 (reference)	< 0.001	-	-	-	-
≥ 55	74	82.4	11.34 (4.96–25.93)	-	-	-	-	-
TERT mutation	-	-	-	-	-	-	-	-
Wild-type	350	98.0	1.00 (reference)	< 0.001	1.00 (reference)	0.008	1.00 (reference)	< 0.001
Mutant	43	67.4	24.15 (10.56–55.25)	-	4.21 (1.46–12.21)	-	9.75 (3.62–26.30)	-
BRAF mutation	-	-	-	-	-	-	-	-
Wild-type	117	92.3	1.00 (reference)	0.331	-	-	-	-
Mutant	199	97.0	0.64 (0.26–1.57)	-	-	-	-	-
Histologic type	-	-	-	-	-	-	-	-
PTC	327	96.6	1.00 (reference)	0.004	1.00 (reference)	0.031	1.00 (reference)	0.009
FTC	66	84.8	3.15 (1.44–6.88)	-	3.04 (1.11–8.34)	-	3.23 (1.35–7.75)	-
Multifocality	-	-	-	-	-	-	-	-
Absent	286	94.4	1.00 (reference)	0.847	-	-	-	-
Present	107	95.3	0.92 (0.39–2.17)	-	-	-	-	-
Lymph node metastasis	-	-	-	-	-	-	-	-
Absent	199	94.0	1.00 (reference)	0.964	-	-	-	-
Present	193	95.3	1.02 (0.47–2.20)	-	-	-	-	-
Extrathyroidal extension	-	-	-	-	-	-	-	-
Absent	352	96.0	1.00 (reference)	< 0.001	1.00 (reference)	0.212	-	-
Present	41	82.9	4.78 (2.14–10.64)	-	1.79 (0.72–4.48)	-	-	-
Distant metastasis	-	-	-	-	-	-	-	-
Absent	370	96.8	1.00 (reference)	< 0.001	1.00 (reference)	0.434	-	-
Present	23	60.9	10.85 (4.86–24.22)	-	1.55 (0.52–4.67)	-	-	-
Stage at diagnosis[e]	-	-	-	-	-	-	-	-
I	329	97.6	1.00 (reference)	< 0.001	-	-	1.00 (reference)	0.067
II	48	85.4	10.42 (4.19–25.92)	< 0.001	-	-	2.87 (0.90-9.11)	0.074
III & IV	16	62.5	26.82 (10.03–71.70)	< 0.001	-	-	4.12 (1.23–13.80)	0.021
Tumor size, cm	-	-	-	-	-	-	-	-
< 2.0	45	95.6	1.00 (reference)	0.014	1.00 (reference)	0.848	-	-
2.0–4.0	293	95.9	1.24 (0.28–5.38)	0.776	0.80 (0.17–3.66)	0.769	-	-
> 4.0	55	87.3	3.96 (0.86–18.34)	0.078	1.03 (0.19–5.69)	0.970	-	-
RAI total dose, mCi	-	-	-	-	-	-	-	-
Per 1 mCi	393	-	1.0033 (1.0024–1.0043)	< 0.001	1.0015 (1.0003–1.0027)	0.013	1.0014 (1.0004–1.0025)	0.009

Abbreviations: *TERT*, telomerase reverse transcriptase; *BRAF*, v-Raf murine sarcoma viral oncogene homolog B; DTC, differentiated thyroid cancer; PTC, papillary thyroid cancer; FTC, follicular thyroid cancer; RAI, radioactive iodine; [a] The number based on available data for a particular variable in the univariate analysis.; [b] Model in which all predictors with univariate *p* values ≤ 0.20 were included; no interactions were considered.; [C] Restricted model, which includes "*TERT*" and "Stage at diagnosis"; no interactions were considered.; [d] Multivariate Cox regression analysis results for "Age" were analyzed and presented for the continuous linear variable.; [e] Staging according to the American Joint Committee on Cancer Thyroid Cancer Staging System, 8th edition, 2016.

Table S4. Associations between *TERT* mutation status or clinicopathological variables (including total RAI dose) and thyroid cancer-specific survival in patients with PTC.

Variables	N[a]	10-year survival rate (%)	Univariate Cox models		Multivariate Cox model 1[b] (extended model)		Multivariate Cox model 2[c] (restricted model)	
			HR (95% CI)	*p*-value	HR (95% CI)	*p*-value	HR (95% CI)	*p*-value
Sex	-	-	-	-	-	-	-	-
Female	276	97.5	1.00 (reference)	0.026	1.00 (reference)	0.244	1.00 (reference)	0.187
Male	51	92.2	3.09 (1.14–8.36)	-	1.99 (0.63–6.29)	-	2.10 (0.70–6.35)	-
Age, years[d]	-	-	-	-	-	-	-	-
Per 5 years	327	-	1.73 (1.42–2.10)	< 0.001	1.37 (1.03–1.81)	0.030	-	-
< 55	265	98.1	1.00 (reference)	< 0.001	-	-	-	-
≥ 55	62	90.3	11.00 (3.87–31.23)	-	-	-	-	-
TERT mutation	-	-	-	-	-	-	-	-
Wild-type	295	99.0	1.00 (reference)	< 0.001	1.00 (reference)	0.009	1.00 (reference)	< 0.001
Mutant	32	75.0	36.27 (11.81–111.42)	-	7.72 (1.68–35.43)	-	17.69 (4.67–66.92)	-
BRAF mutation	-	-	-	-	-	-	-	-
Wild-type	65	98.5	1.00 (reference)	0.253	-	-	-	-
Mutant	199	97.0	3.31 (0.42–25.89)	-	-	-	-	-
Multifocality	-	-	-	-	-	-	-	-
Absent	228	96.9	1.00 (reference)	0.664	-	-	-	-
Present	99	96.0	1.25 (0.46–3.37)	-	-	-	-	-
Lymph node metastasis	-	-	-	-	-	-	-	-
Absent	135	97.0	1.00 (reference)	0.410	-	-	-	-
Present	191	96.3	1.56 (0.54–4.49)	-	-	-	-	-
Extrathyroidal extension	-	-	-	-	-	-	-	-
Absent	290	97.6	1.00 (reference)	0.002	1.00 (reference)	0.174	-	-
Present	37	89.2	4.67 (1.73–12.65)	-	2.20 (0.71–6.85)	-	-	-
Distant metastasis	-	-	-	-	-	-	-	-
Absent	313	97.4	1.00 (reference)	0.007	1.00 (reference)	0.261	-	-
Present	14	78.6	5.59 (1.61–19.47)	-	2.34 (0.53–10.28)	-	-	-
Stage at diagnosis[e]	-	-	-	-	-	-	-	-
I	274	98.2	1.00 (reference)	< 0.001	-	-	1.00 (reference)	0.324
II	42	92.9	9.60 (3.05–30.25)	< 0.001	-	-	1.90 (0.48–7.52)	0.358
III & IV	11	72.7	31.06 (8.97–107.58)	< 0.001	-	-	3.11 (0.71–13.70)	0.134
Tumor size, cm	-	-	-	-	-	-	-	-
< 2.0	35	97.1	1.00 (reference)	0.087	1.00 (reference)	0.910	-	-
2.0–4.0	253	97.2	1.53 (0.20–11.84)	0.685	0.99 (0.12–8.27)	0.992	-	-
> 4.0	39	92.3	4.70 (0.55–40.19)	0.158	1.32 (0.13–13.87)	0.818	-	-
RAI total dose, mCi	-	-	-	-	-	-	-	-
Per 1 mCi	327	-	1.0031 (1.0017–1.0044)	< 0.001	1.0014 (1.0000–1.0028)	0.047	1.0014 (1.0000–1.0027)	0.044

Abbreviations: *TERT*, telomerase reverse transcriptase; *BRAF*, v-Raf murine sarcoma viral oncogene homolog B; PTC, papillary thyroid cancer; FTC, follicular thyroid cancer; RAI, radioactive iodine; [a] The number based on available data for a particular variable in the univariate analysis.; [b] Model in which all predictors with univariate *p* values ≤ 0.20 were included; no interactions were considered.; [c] Restricted model, which includes "*TERT*" and "Stage at diagnosis"; no interactions were considered.; [d] Multivariate Cox regression analysis results for "Age" were analyzed and presented for the continuous linear variable.; [e] Staging according to the American Joint Committee on Cancer Thyroid Cancer Staging System 8th edition, 2016.

Copyright: © 2021 by the authors. Licensee MDPI, Basel, Switzerland. This article is an open access article distributed under the terms and conditions of the Creative Commons Attribution (CC BY) license (http://creativecommons.org/licenses/by/4.0/).
